# pH-responsive chitosan based hydrogels for the simultaneous delivery of atorvastatin calcium and ezetimibe

**DOI:** 10.1039/d6ra05028j

**Published:** 2026-07-23

**Authors:** Naba Shabbir, Waqar Siddique, Humayun riaz, Muhammad Zaman, Hamoud Alotaibi, Mona Al Hamod, Saleh Alfuraih, Noura Al Hamood

**Affiliations:** a Riphah Institute of Pharmaceutical Sciences, Riphah International University Lahore Campus 54000 Lahore Pakistan wpharmacist@gmail.com naba.shabbir34@gmail.com; b Rashid Latif College of Pharmacy, Rashid Latif Medical Complex Lahore Pakistan humayun.riaz@rlmc.edu.pk; c Faculty of Pharmaceutical Sciences, University of Central Punjab Lahore Pakistan m.zaman2157@gmail.com; d Department of Pharmaceutics, Faculty of Pharmacy, Northern Border University Rafha 73213 Saudi Arabia Hamoud.Alotaibi@nbu.edu.sa Mona.Alhumodh@nbu.edu.sa; e Department of Pharmacology and Toxicology, Faculty of Pharmacy, Northern Border University Rafha 76313 Saudi Arabia Saleh.Alforih@nbu.edu.sa; f Department of Pharmaceutics, Faculty of Pharmacy, King Khalid University Abha 62529 Saudi Arabia nhamud@kku.edu.sa

## Abstract

The management of cholesterol by using Atorvastatin calcium (ATV) and Ezetimibe (EZE) is often restricted by the fact that they belong to BCS Class II drugs, thus, low aqueous solubility and low systemic bioavailability. The research has created a new pH-responsive hydrogel involving a natural polycationic polysaccharide, chitosan, to increase residence time in the sites of absorption and offer controlled, concurrent delivery of both drugs. The hydrogels were synthesized through a free-radical polymerization process, which entailed dissolving chitosan in aqueous acetic acid, initiation by ammonium persulfate (APS) and addition of acrylic acid (AA) and *N*,*N*′-methylene bisacrylamide (MBA) in drops to create a stable 3D cross-linked structure. The numerical optimization was performed with Design Expert software, in which the ratios of components are accurately tuned with the help of the use of the poly equations and 3D response surface plots. This methodology removed hit and trial error leading to a maximized formulation in which the real experimental values, including 87.98% porosity and 98.76% gel fraction, were in good agreement with the software predictions, which confirmed the strength of the design. Findings showed that the internal structure was greatly determined by the formulation parameters; the higher the monomer concentrations, the better the porosity (97.53%) and the gel fraction (97.79%). The system was also very pH-sensitive as it swelled little in acidic environment (pH 1.2) but swelled the most (187.71%) in simulated intestinal fluids (pH 6.8 and 7.2) which allowed the system to dissolve 91.99% of ATV and 90.77% of EZE within 24 h. This mathematically designed platform with dual drug delivery is designed to overcome the drawbacks of a single-drug matrix for cholesterol management and prevent premature leakage from the stomach.

## Abstract 2

The management of cholesterol by using Atorvastatin calcium (ATV) and Ezetimibe (EZE) is often restricted by the fact that they belong to BCS Class II drugs, thus, low aqueous solubility and low systemic bioavailability.

## Graphical abstract

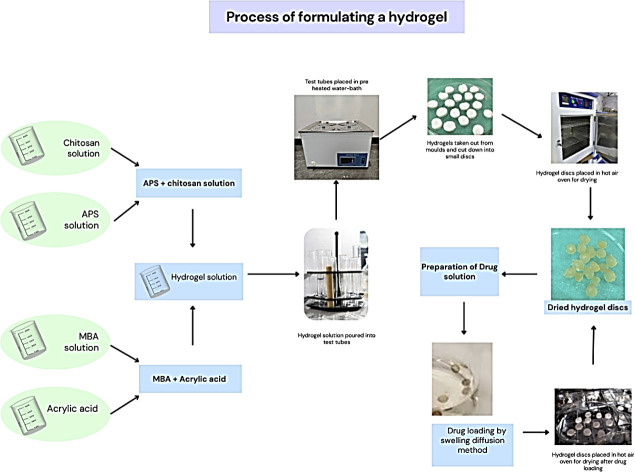

## Introduction

Hydrogels are cross-linked polymeric networks, which have the special property of being able to absorb and store large amounts of water or any biological fluid without dissolving. Hydrogels have been used as intelligent carriers in the area of drug delivery. They are designed to change in response to external stimuli, including most prominently pH, temperature, or ionic strength, through changing their size or the length of their network.^1^ The cross-linking density determines the architecture of a hydrogel. Covalent bonding is used to maintain structure in a chemical hydrogel, whereas a physical hydrogel is based on hydrogen bonding or entanglement of molecules. In oral administration, pH-sensitive hydrogels are especially useful, as they can remain in intact form in the acidic environment of the stomach in order to protect the drug and to swell in the alkaline environment of the small intestine to liberate the loaded drug at the site of action for optimal absorption.^2^

ATV is a synthetic lipid-lowering drug that is categorized under the group of drugs known as statins. It competitively inhibits the rate-limiting enzyme of 3-hydroxy-3-methylglutaryl-coenzyme A (HMG-CoA) reductase in the conversion of HMG-CoA to cholesterol precursor mevalonate.^3^ Although ATV is very effective in the process of lowering LDL cholesterol and triglycerides, the drug belongs to the Biopharmaceutical Classification System (BCS) as a Class II drug. This implies that it is highly permeable and has a very low aqueous solubility. Its oral bioavailability is further undermined by high first-pass hepatic and gut-wall metabolism, resulting in a systemic bioavailability of approximately 12% only.^4^

EZE belongs to the class of cholesterol absorption inhibitors. EZE binds to the brush border of the small intestine and inhibits the uptake of cholesterol through the Niemann–Pick C1-Like 1 (NPC1L1) protein 5. This minimizes the supply of intestinal cholesterol to the liver. EZE, like ATV, is a BCS Class II drug. It is insoluble in water, resulting in unpredictable absorption and marked inter-patient variability of therapeutic response. As a combination, ATV and EZE are a combination of two different processes, the synthesis and the absorption of cholesterol, which is often required in patients with severe hypercholesterolemia.^6^

Chitosan is a natural polycationic polysaccharide that is produced as a result of the deacetylation of chitin. It is being used in biomedical practices extensively because of its biodegradability, biocompatibility, and non-toxicity.^7^ In acidic to neutral pH, the primary amine groups in the chitosan backbone get positively charged, and thus they are able to establish an electrostatic interaction with the negatively charged sialic acid residues in the gastrointestinal mucus.^8^ The effect of this interaction is that the residence time of the dosage form at the absorption site is increased, which may overcome the high transit times that can usually limit absorption of poorly soluble drugs.^9^

One of the important clinical objectives is the simultaneous administration of ATV and EZE on a single-dose basis, but the low solubility of the two drugs creates a formulation challenge.^10^ Conventional tablet preparations do not offer the progressive, portioned release to obtain the most positive synergistic impact of these two medications.^11^ Current research work is novel, in fact, due to the development of a chitosan (acrylic acid) hydrogel developed with the specific aim of loading these agents dually.^12^ In the current study, the aim was to dissolve the drugs in the hydrophilic polymeric mesh to transform the crystal structure to an amorphous one, therefore, improving their dissolution rate.^13^ Gastric protection, the acid responsiveness of acrylic acid–chitosan copolymer is such that the drugs are not subjected to the acidic gastric juice, and consequently, they are not released early. Site-Specific delivery, the system can be employed to deliver drugs *via* the intestinal wall when a local and controlled release profile is required.^14^ The system is to be used with the network of chitosan. This multi-functional strategy is a major development compared to traditional lipid-lowering therapy, and it may provide a possible improvement in decreasing the dosing frequency.

For drugs that belong to BCS Class II (lipid-lowering agents such as ATV and EZE), whose physiological properties are lipophilic and exist in the form of poorly soluble form, there have been several advanced drug delivery systems (DDS) developed. Nanoemulsions of lipids have been used extensively to convert lipophilic formulations into fine oil in water nano-emulsion in the gastrointestinal tract, and thus avoiding the first-pass effect of the liver. Lipid-based nanocarriers, such as self-nanodrug delivery systems (SNEDDS).^15^ In the same way, nanostructured lipid carriers (NLCs) have been optimized to encapsulate ATV in an amorphous form in a biocompatible lipid matrix, which greatly lowers serum lipid levels.^16^ In addition to lipid nanostructures, solid-state engineering methods, such as cyclodextrin inclusion complexes, have been developed with natural β-cyclodextrin and other synthetic derivatives that form a protective hydrophobic core, leading to an exponentially increased wetting and solubility of EZE.^17^ Furthermore, multiparticulate systems and oral films, which are small particles coated with a shell of micellar EZE and statin, have been shown to be promising approaches for the simultaneous delivery of statin and micellar EZE for expedited absorption in the mucosal or gastrointestinal tract.^18^ However, they are often not sufficient to provide the structural macro-protection necessary to avoid early gastric leakage, while being used to enhance pure thermodynamic solubility. The hydrogel developed in this work is a pH-responsive polyanionic mesh that undergoes an electrostatic relaxation under intestinal conditions, which is crucial for the targeted and simultaneous delivery.

The developed chitosan-*g*-poly(acrylic acid) hydrogel is a combination of two mechanisms, namely, the physicochemical and physiological mechanisms, which could enhance the poor solubility and low bioavailability of ATV and EZE.^19^ The highly wettable amorphous state of the drugs in the hydrophilic matrix results in an extremely low thermodynamic barrier to dissolution and the prevention of recrystallization during swelling. At the same time, the chitosan backbone properties create a slower release of the product in the intestine and increase the drug concentration at the intestinal wall in the form of a supersaturated concentration gradient,^20,21^ which may result in a higher percentage of the drug absorbed into the bloodstream than the drug from the free crystal combination^22–26^

Design-Expert is a statistical software package specifically created to assist researchers in applying Design of Experiments (DOE) methodology. Its primary function is to optimize products and processes by helping users plan, execute, and analyze experiments efficiently. It enables scientists to efficiently design various experiments, including Factorial, Response Surface Methodology (RSM), and Mixture designs, thereby systematically varying multiple process factors to understand their main and interactive effects on desired outcomes. The software provides robust statistical analysis, particularly ANOVA, and powerful visualization tools like 2D contour plots and 3D response surface plots, which are critical for identifying optimal conditions, modeling complex relationships, and effectively communicating optimization results in a research or industrial setting.^27^

The concentrations of chitosan, acrylic acid, MBA, and APS are the independent variables. The responses, such as swelling behavior, drug entrapment efficacy, gel fraction, and *in vitro* drug release, are considered dependent variables. By increasing the concentration of monomer, swelling was increased. Increased concentration of crosslinking causes stronger crosslinking between polymer and monomer, resulting in a decrease in the swelling of the hydrogel.

Cardiovascular diseases (CVDs) are still the top cause of death in the world, impacting more than 600 million people and affecting over 200 million statin-treated patients; however, up to 70% of high-risk individuals are not achieving the target LDL-C level with statin monotherapy alone. ATV and EZE have a complementary dual mechanism leading to a more effective lipid-lowering effect. But the multi-pill dosing or standard pill is poorly accepted by patients, has a rapid drug burst, and variable intestinal absorption. This is the novelty of the work, to develop a dual-compartment, chitosan-*g*-PAA hydrogel macro-matrix as compared to the traditional tablets, fast dissolving films, and nanocarriers. This hydrogel platform enables swelling-controlled co-release, prolongs intestinal contact time, and sustains a supersaturation gradient within a localized region, overcoming the issues of low drug loading and manufacturing bottlenecks of nano-systems and rapid disintegration of traditional tablets. This greatly improves the oral absorption, amorphous drug stability, ease of daily dosing, and direct increase in patient compliance for disease management. By employing RSM, Authors successfully mapped and optimized the non-linear multi-factorial relationships between crosslinking density, swelling ratio, and dual-drug loading efficiency. This yields a robust, highly scalable, and reproducible hydrogel platform specifically optimized for intestinal co-delivery.

## Materials and methodology

### Materials and chemicals

Acrylic acid (AA), *N*,*N*′-methylene bisacrylamide (MBA), ammonium persulfate (APS), and acetic acid was purchased from Sigma Aldrich, US. ATV and EZE were provided as gift samples from Shrooq Pharmaceuticals. All of the chemicals involved in the current research work are of analytical grade.

### Method of preparation

**Fig. 1:**
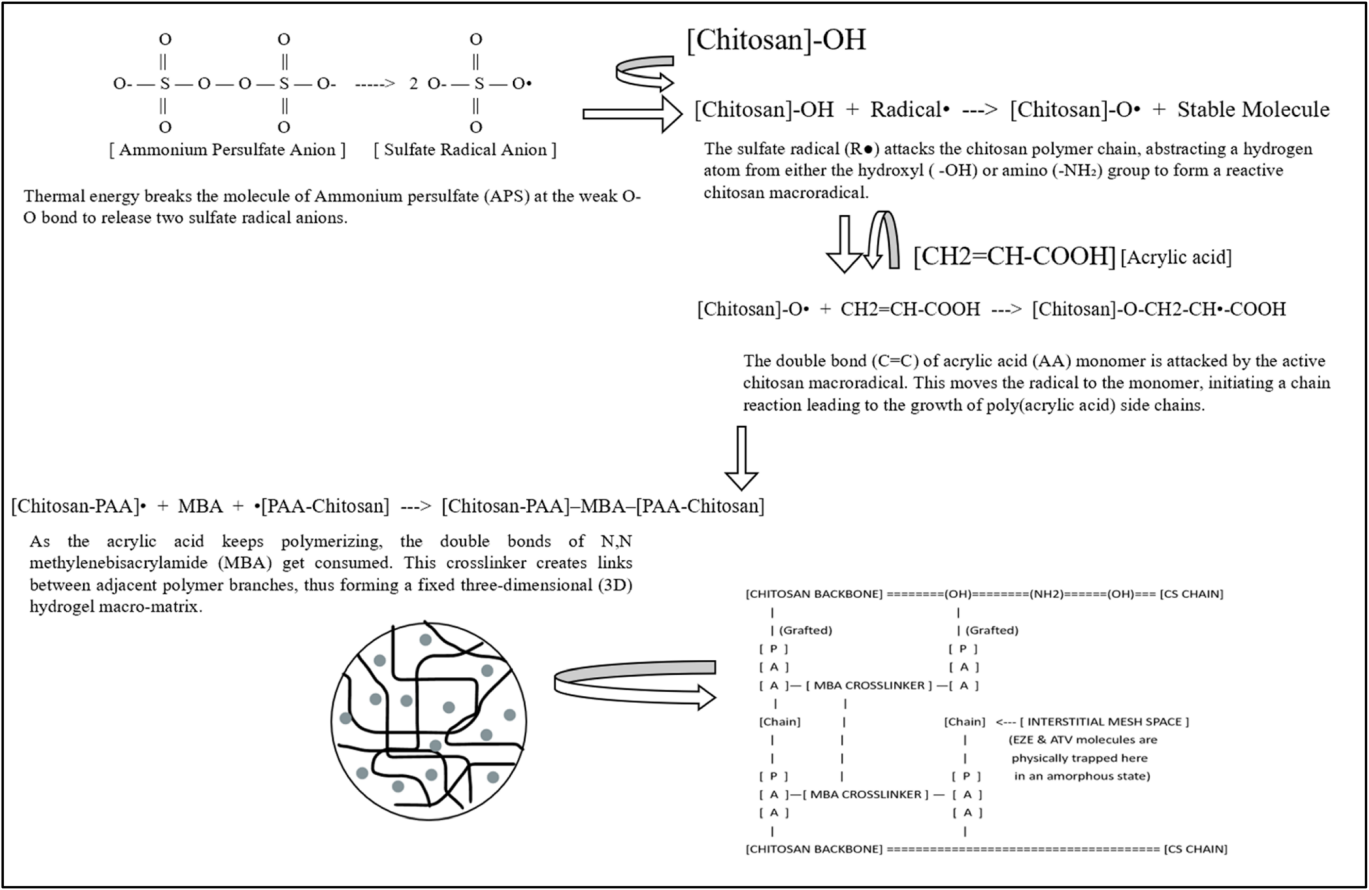
Schematic presentation of the preparation of hydrogels.

The simultaneous hydrophobic drug loading design (Table 1) was accommodated by modifying the polymeric matrix, which was adapted from established cross-linking methodologies, including free radical polymerization technique.^28^ In this case, a 1.0% (v/v) aqueous acetic acid solution was used to dissolve chitosan (190–310 kDa, 75–85% deacetylation). This mixture was magnetically stirred for four hours at room temperature (25 °C) to obtain a clear and homogeneous (w/v) polymer solution that was hydrophilic. In order to start the polymerization, an aqueous solution of APS was introduced into the chitosan solution. The step is of prime importance because the APS is a free-radical initiator, which forms active sites along the chitosan chains at which the monomer is able to graft easily. The monomer phase was then prepared in a different flask, whereby AA was mixed with the cross-linking material, and MBA was dissolved in deionized water. The subsequently prepared secondary solution was added dropwise to the chitosan solution that had already been initiated under steady mechanical stirring to make sure that the cross-linker and the monomer were distributed evenly across the matrix of the polymer. To remove any trapped air and to have a thick, homogenous inner structure, the reaction mixture was sonicated for a few minutes. The homogeneous mixture was subsequently transferred into special glass molds and put in a water bath with the temperature controlled at 60 °C for 3 hours. This heat energy enables the reaction of the cross-linking process to occur, and the liquid mixture converts to a solid hydrogel (Fig. 1).

**Table 1:** Formulation design of EZE and ATV hydrogels by using RSM

S. no.	Chitosan (cross linker) (g)	Acrylic acid (monomer) (mL)	MBA (crosslinker) (g)	APS (initiator) (g)
1	0.02	3.25	3.25	0.02
2	0.01	3.25	2.5	0.02
3	0.02	4.5	4	0.02
4	0.02	3.25	4	0.02
5	0.03	3.25	3.25	0.02
6	0.01	2	3.25	0.02
7	0.02	3.25	3.25	0.02
8	0.03	4.5	3.25	0.02
9	0.03	4.5	2.5	0.02
10	0.02	2	4	0.02
11	0.02	3.25	2.5	0.02
12	0.02	2	2.5	0.02
13	0.01	4.5	3.25	0.02
14	0.01	3.25	4	0.02
15	0.03	2	3.25	0.02
O.F	0.01	4	3.25	0.02

Design-Expert is a statistical software package specifically created to assist engineers and scientists in applying Design of Experiments (DOE) methodology. It enables scientists and engineers to efficiently design various experiments, including Factorial, RSM, and Mixture designs, thereby systematically varying multiple process factors to understand their main and interactive effects on desired outcomes. For this purpose, RSM with a Box–Behnken Design (BBD) consisting of three factors was used to model and optimize the formulation parameters of the chitosan-*g*-PAA hydrogel macro-matrix (Table 1). BBD is an independent rotatable quadratic response surface design for the study of the non-linear interactive effects of critical formulation variables such as polymer concentration, monomer ratio, and crosslinker density on important performance parameters like drug loading efficiency and swelling index. This method allows for a substantial decrease in the number of experimental trials, while simultaneously producing high-precision polynomial predictive models. This system offers a mathematically validated framework for attaining an optimum balance between matrix swelling capacity, dual-drug entrapment, and controlled intestinal release kinetics. The software gives us good statistical analysis capability, especially Analysis of variance (ANOVA) and high-quality visualization functions such as 2D contour plots and 3D response surface plots, which are vital in determining the optimal conditions, modeling results of complex relationships, as well as presenting the results of optimization in an effective manner.

After this process, the hydrogels were removed from the molds and sliced into homogeneous 2 mm discs. The discs were washed in ethanol-distilled water to remove any remaining monomers, initiator residues, or non-crosslinked chains of polymer. Lastly, the dried discs of hydrogel were placed in a hot air oven at 40 °C until they attained a uniform weight to produce the final crosslinked matrix that will be loaded with drugs and characterized.

### Characterization techniques

#### Fourier transform infrared spectroscopy (FTIR)

The Fourier Transform Infrared Spectroscopy (FTIR) technique is used to analyze the functional groups. The chemical purity of the active pharmaceutical ingredients (API) and excipients in the powder, as well as in formulation form, was verified by recording FTIR spectra. Potassium bromide (KBr) was added to the samples to form clear pellets and scanned between 4000 and 400 cm^−1^.

### Thermal and crystallographic analysis

#### Differential scanning calorimetry (DSC)

Samples were heated in an aluminum pan from 30 °C to 300 °C with a heating rate of 30 °C min^−1^ in the presence of a nitrogen atmosphere to monitor melting and glass transition temperatures.

#### Powder X-ray diffraction (PXRD)

To ascertain the physical form (crystalline or amorphous) of the drugs in the formulation, the samples were put through an X-ray diffractometer using radiation over the wavelength of 5° to 60 °C.

### Sol–gel fraction and swelling behavior

Fraction of hydrogel discs dried by sol–gel was extracted by simple immersion in distilled water over 48–72 hours to eliminate the uncross-linked fraction (sol). The discs were dried again and measured to determine the content of the gel. It is demonstrated that swelling is sensitive to pH. Swelling studies were performed by placing dry hydrogel discs in buffers of varying pH (1.2, 6.8, and 7.2) to simulate gastric and intestinal environments. At intervals, the weight of the swollen discs was recorded until they attained the equilibrium.^29^

### Porosity

The solvent replacement method was used to measure the porosity of the hydrogels. Dry discs were pre-weighed and were left in an absolute ethanol solution over a 24-hour period. The discs were then weighed, wiped, and removed. The percentage of pore space was calculated using the increase in weight.^30^

### Efficiency of drug loading and entrapment

The swelling diffusion method was used in the process of drug loading. Dry hydrogel discs were subjected to a concentrated solution of drug (1% w/v) of ATV and EZE in an appropriate solvent system. The swelling of the discs was allowed until the drugs were absorbed into the matrix, and then the discs were dried. The entrapment efficiency was determined by removing the drugs from the loaded discs into a buffer and measuring them with the use of UV spectroscopy or HPLC (eqn (1)).^31^

1 EE (%) = (*W* initial drug − *W* free drug)/*W* initial drug × 100

### *In vitro* dissolution and RP-HPLC

A USP Type II (paddle) apparatus at 50 rpm was used to conduct dissolution studies in 900 mL of 1.2 pH and 7.2 pH buffer. Withdrawal of samples and analysis were conducted in intervals, and analyzed by a validated RP-HPLC technique. The mobile phase comprised 28 : 72 (v/v) of acetonitrile and ammonium acetate, run with a flow rate of 1.0 mL min^−1^, through a C18 column, with detection at 242 nm.

## Results

### Calibration curves of analysis

The standard calibration curves of the two APIs were prepared in a UV spectrophotometer to determine the accurate measurement of the drug concentrations that were subsequently used in the loading of the drugs.^32^ The ATV and EZE were dissolved in 70% methanol, and their respective maximum wavelengths were 245 nm and 232 nm.^33,34^ The curves that appeared were of good linear correlation between the concentration and the absorbance, and the regression coefficient (*R*^2^) of the two drugs was 0.999 for EZE and ATV, respectively. This good linearity gives confirmation that the analysis method is very accurate, and it can be used to compute the amount of medication picked and discharged (Table 2).

**Table 2:** Standard curve data for ATV (245 nm) and EZE (232 nm) UV spectrophotometer

Concentration µg ml^−1^	Absorbance of ATV at 245 nm	Absorbance of EZE at 232 nm
4	0.455	0.455
8	0.555	0.555
12	0.665	0.665
16	0.763	0.763
20	0.876	0.876

### Fourier transform infrared spectroscopy (FTIR)

**Fig. 2:**
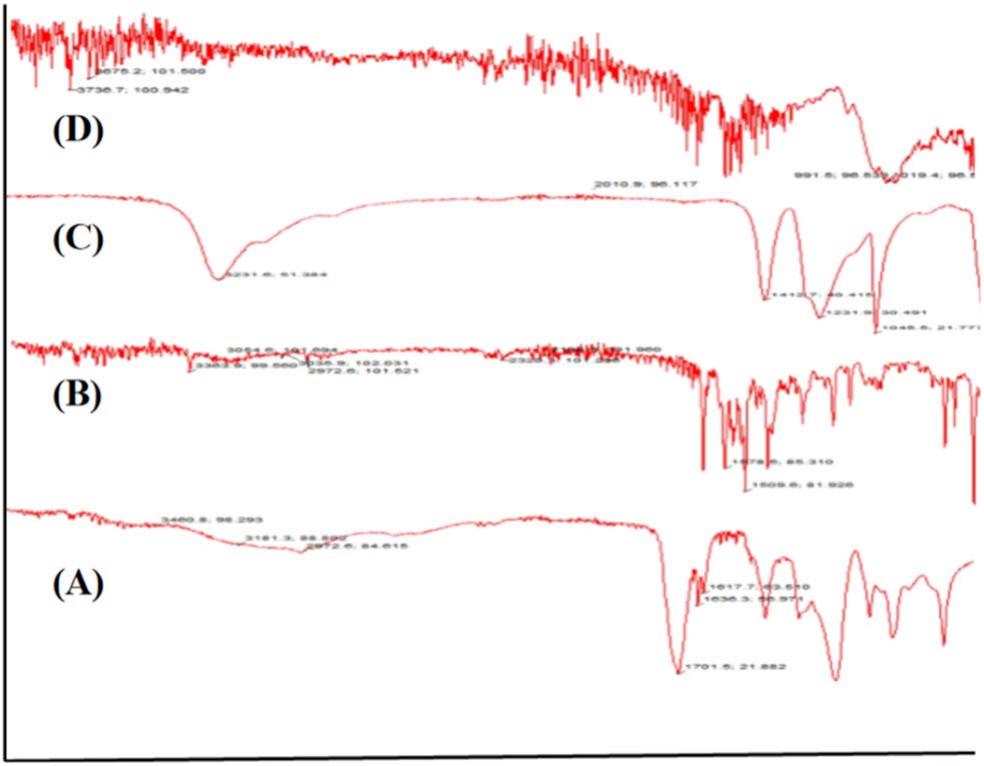
FTIR spectra of (A) acrylic acid, (B) ATV, (C) APS, (D) chitosan.

**Fig. 3:**
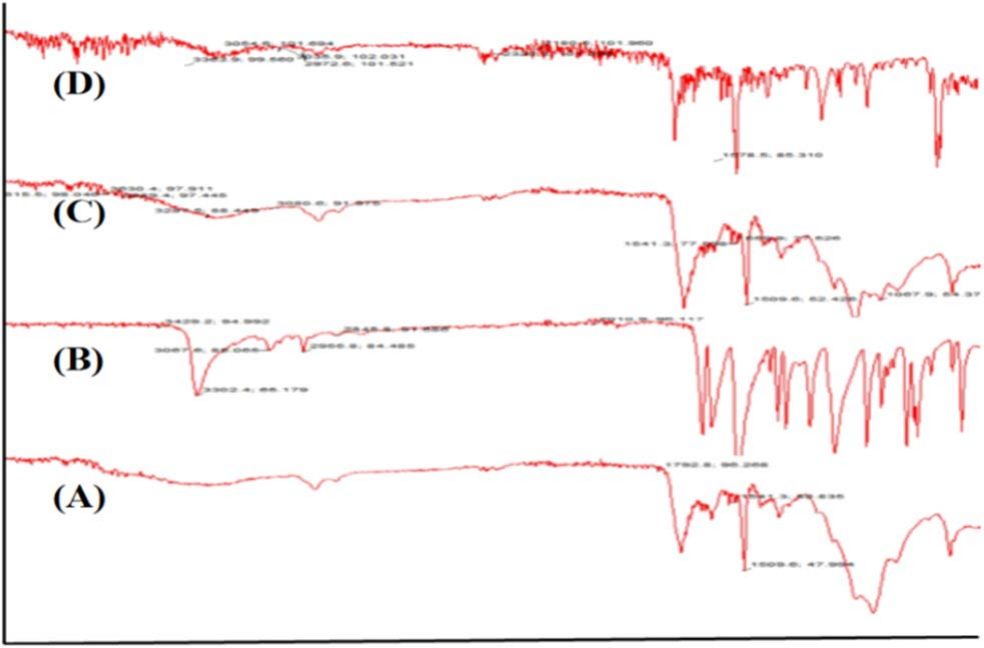
FTIR spectra of (A) drug-loaded hydrogels, (B) un-loaded hydrogels, (C) MBA, (D) EZE.

The formation of the polymeric network and the existence of the characteristic functional groups of all components were confirmed by FTIR analysis.^35^ The chitosan spectrum produced strong O–H and N–H stretches (3621.1 to 3295.0 cm^−1^), whereas the acrylic acid spectrum had strong carbonyl (C=O) vibrations (1772.3 to 1700 cm^−1^). The appearance of a distinct peak at 1792.8 cm^−1^ in drug-loaded hydrogels was a specific indication of loading EZE because this peak is attributed to its typical 2-lactam ring (Fig. 2 and 3).^36,37^

FTIR analysis showed the covalent cross-linking of the hydrophilic polymer scaffold to be successful and that the encapsulated drugs were chemically stable. The spectrum of chitosan was characterized by a broad –OH and primary –NH_2_ stretching band (3500–3200 cm^−1^) and a distinct amide II (N–H bending) peak near 1590 cm^−1^. The band of amide II showed a significant hypochromic shift, intensity decrease, and a new sharp covalent stretching vibration after the crosslinking. This transition is an indication of the transformation of primary amino groups into stable cross-linked linkages, similar to what was observed in Fig. 2.^38^ Moreover, the structure of both lipophilic drugs, amide carbonyl (C=O) stretching for ATV and β-lactam ring carbonyl stretching for EZE, was clearly observed (Fig. 3) and retained in the dual cargo hydrogel. The subtle broadening and shifting of these peaks without the appearance of anomalous bands illustrate that ATV and EZE were successfully hosted in the hydrophilic mesh of the macropores by weak non-covalent interactions (*e.g.*, hydrogen bonding) and not by degradation of the ATV molecules, consistent with co-amorphous multi-drug architectures developed for synchronized lipid-lowering therapies.^39^

### PXRD and DSC/TGA

**Fig. 4:**
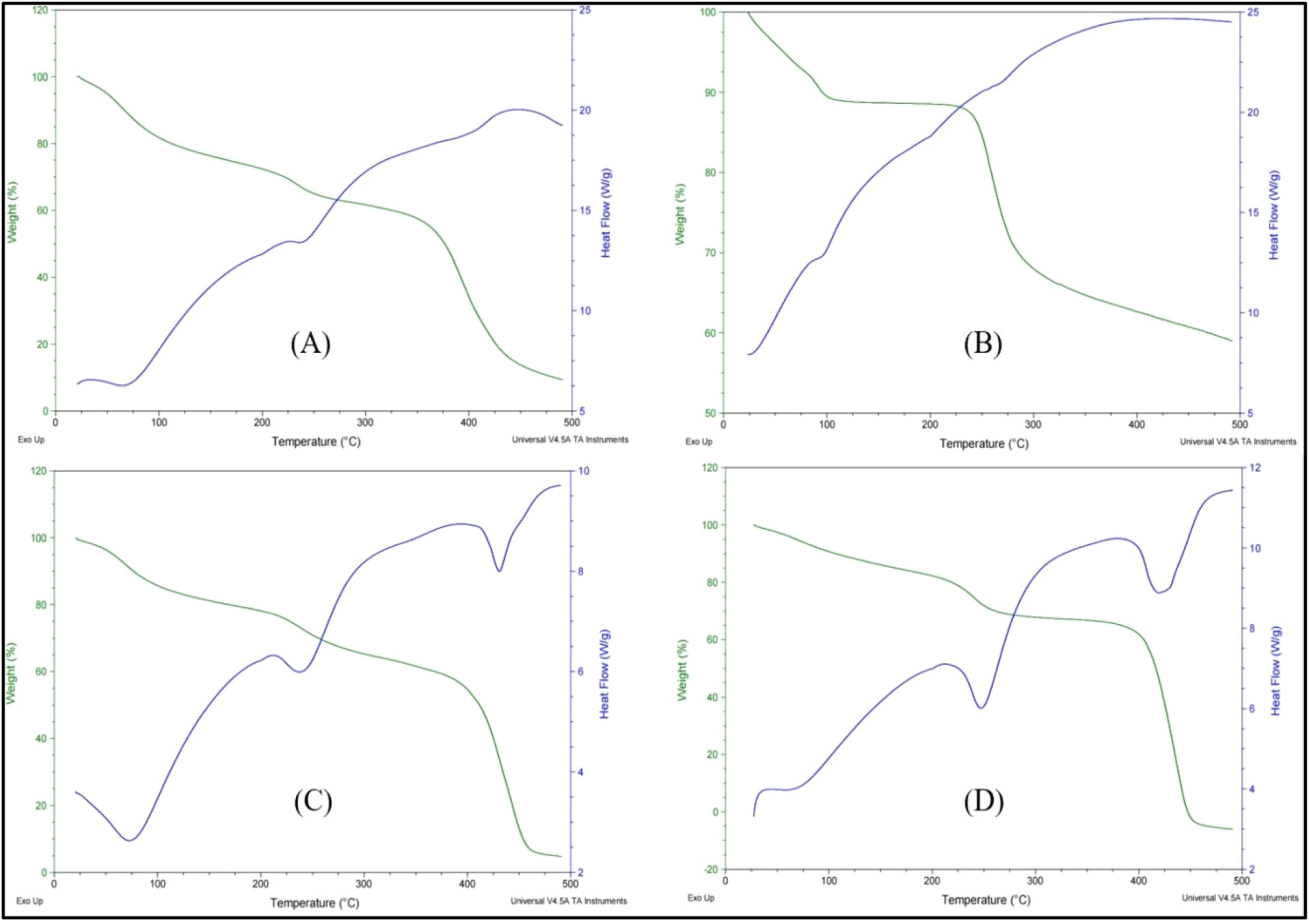
Showing the results of DSC/TGA of ATV (A), EZE (B), unloaded hydrogel (C) and loaded hydrogel (D). Blue line indicates DSC and green shows the results of TGA.

**Fig. 5:**
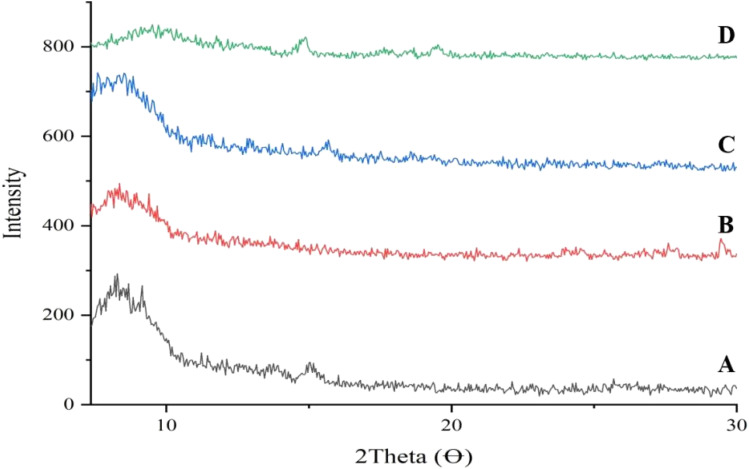
PXRD curves of (A) ATV, (B) EZE, (C) drug-loaded hydrogel, (D) unloaded hydrogel.

Thermo-gravimetric analysis was the evidence of a physical change in the drugs. In pure ATV and EZE, sharp peaks and clear melting points (around 150 °C and 160 °C, respectively) were visible, and the two products were highly crystalline, but in the hydrogel formulation, none of these features were present at all or were much less evident. This shows that the drugs were successfully converted to an amorphous form or molecularly dispersed in the polymeric mesh, which is a very significant parameter in improving the solubility and bioavailability of BCS Class II drugs (Fig. 4 and 5).^40–43^

The disappearance of crystallinity in the PXRD overlays in the crosslinked chitosan-*g*-PAA hydrogel is quantitatively confirmed by a negligible residue crystallinity index, the amorphization of ATV and EZE.^44^ Even though there are thermodynamic driving forces present during the drying step at 40 °C, the rigid and crosslinked polymeric network provides a strong kinetic barrier to limit the mobility of molecules and prevent phase separation.^45^ This structural immobilization, further strengthened by the massive hydrogen-bonding anchors, effectively prevents the risk of recrystallization, both after the drying and during 24 hours of releasing the drug in a swelling-controlled manner, thus providing a long-term solid-state stability of the co-delivery platform.^46,47^

### Structural integrity and physical appearance

**Fig. 6:**
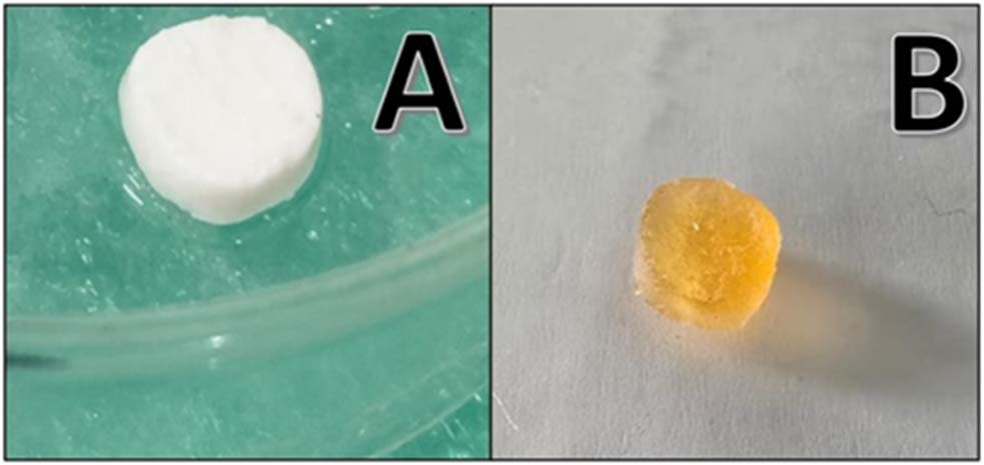
Hydrogel upon formulation (A), hydrogel after drying (B).

At the time of initial formulation, the hydrogels appeared as soft, elastic products with a thick consistency and pale-yellow appearance upon complete drying (Fig. 6). These physical state alterations are an indication of successful cross-linking of the polymer chain into a stable 3D network.^13,48^

### Swelling studies

**Fig. 7:**
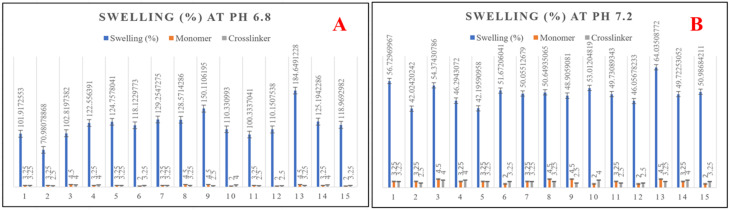
Swelling characteristics of the hydrogel, determined at a constant pH of 6.8 (A) and 7.2 (B), demonstrating the role of monomer and cross-linker concentrations.

Swelling behavior, which is pH-responsive hydrogels, demonstrated a high degree of pH-sensitive swelling, which is a crucial attribute in targeted intestinal delivery.^49^ The acidic conditions that were simulating the stomach (pH 1.2) had a relatively low swelling with ratios ranging between 42.02% and 64.02.^13^ In contrast, the hydrogels demonstrated the maximum expansion under the intestinal conditions (pH 6.8 and 7.2), and their swelling was up to 187.71%. This action makes sure that the drugs are not exposed to the hostile gastrointestinal environment but rather are released at the absorption site in the small intestine (Fig. 7).^50^

### Porosity analysis

**Fig. 8:**
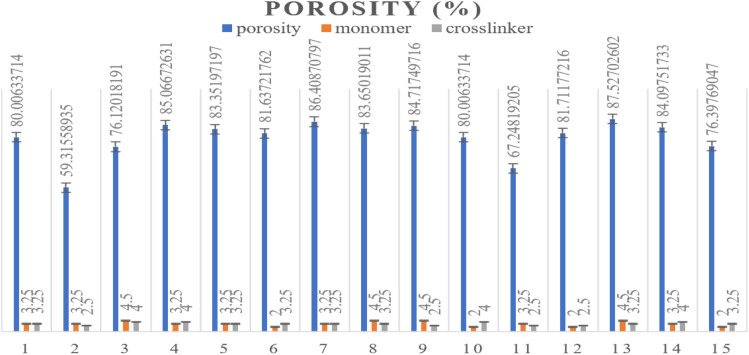
Percentage porosity of hydrogel formulations.

A very important parameter of hydrogels is porosity because it defines the volume within which the drugs can be loaded and the release of the therapeutic factors thereof. The solvent replacement technique was used in the study, and the findings showed that the hydrogels had a macroporous structure whose porosity values were between 59.32 and 87.53% (Fig. 8). The findings have shown a direct relationship between the concentration of the monomers and porosity; with the increase of the concentration of acrylic acid, the internal network became more open and porous.^51,52^ The high porosity has been effective in enabling the hydrogel to take up enormous quantities of the drug solution and the diffusion of ATV and EZE when subjected to intestinal fluids.

### Sol–gel fraction evaluation

**Fig. 9:**
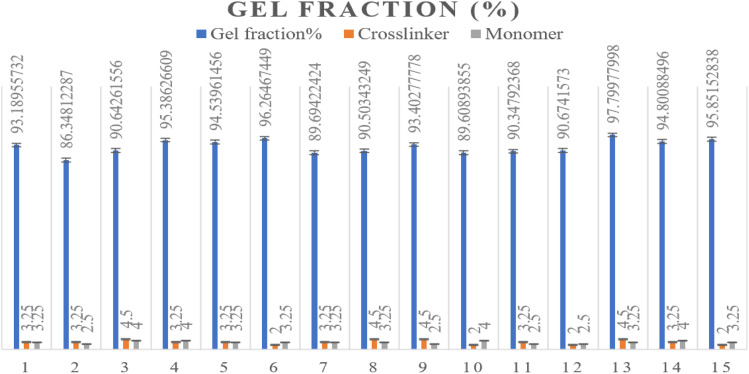
Percentage gel fraction as a function of varying cross-linker and monomer concentrations.

The sol–gel fraction experiment was undertaken to identify the level of cross-linking and the mechanical stability of the formulated polymeric network.^53^ The fraction insoluble in the hydrogel is the so-called gel fraction, whereas the fraction that does not react is the so-called sol fraction that includes unreacted monomers and polymers. The results indicated that it contained a large amount of gel (74.94 to 97.79%). It was noted that the higher the concentration of the monomer, as well as the cross-linker (MBA), the higher the gel fraction (Fig. 9).^54^ It means that a strong and chemically stable 3D scaffold is formed, and it can retain its structural integrity and protect the drug load against the harsh gastric conditions and subsequently deliver it to the target site.^55^

### *In vitro* drug release

The combination-loaded hydrogel formulations (F1 through F15) were used to assess the *in vitro* release kinetics of EZE and ATV over 24 hours. The hydrogel's capacity for extended drug administration was demonstrated by the sustained-release behavior of both medications.

The initial phase of EZE release was fast, with cumulative release in the first hour varying between around 16% (F2) and 29% (F1) (Fig. 11). Release percentages were particularly high by 6 hours, ranging from 39% (F2) to 68% (F1), suggesting a substantial initial release that is frequently ascribed to the drug's proximity to the hydrogel surface. Between 68.36% (F2) and 92.93% (F13), the highest cumulative release at 24 hours differed considerably throughout the formulations. EZE was successfully released throughout the research, as seen by the formulations with the highest overall release, F13, F1, and F8.

**Fig. 10:**
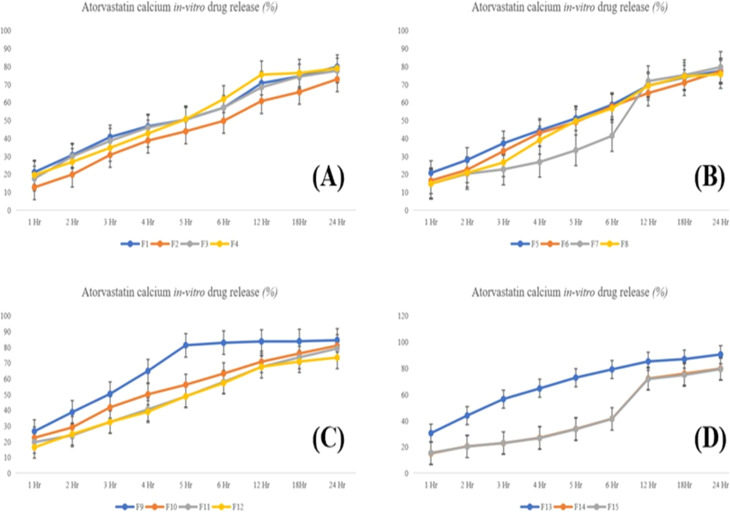
*In vitro* drug release pattern of ATV (A) (F1–F4), (B) (F5–F8), (C) (F9–F12), and (D) (F13–F15) formulations.

In contrast to EZE, the release of ATV likewise displayed a persistent pattern, but often with a delayed initial burst. The range of values of the 1-hour release was 12.82% (F2) to 30.63% (F13) (Fig. 10). The total release varied from 41.13% (F15) to 82.93% (F9) by the 6-hour point. The overall release at 24 hours varied between 72.99% (F2) and 90.59% (F13). The second medication component was effectively delivered by formulations F13, F9, and F4, which showed the highest overall ATV release.^56^

**Fig. 11:**
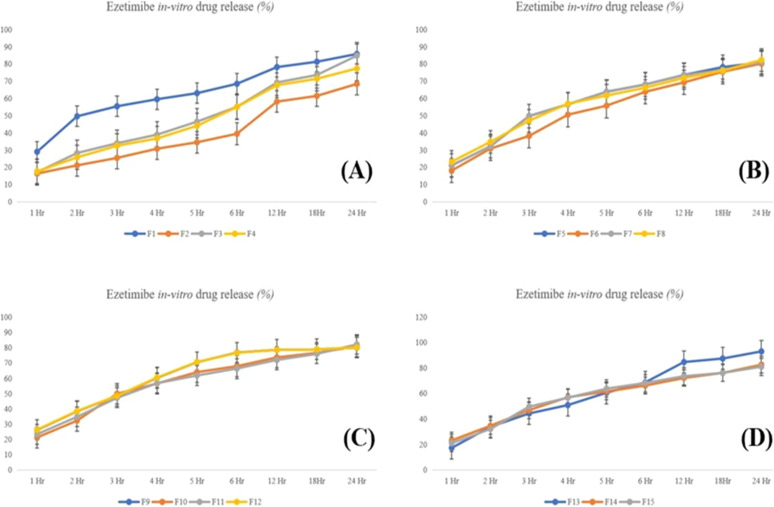
*In vitro* drug release pattern of EZE of (A) (F1–F4), (B) (F5–F8), (C) (F9–F12), and (D) (F13–F15) formulations.

The optimized chitosan-*g*-PAA hydrogels of ATV and EZE were evaluated *in vitro* by a USP Type II (paddle) dissolution apparatus at a constant rotation speed of 50 rpm. The dissolution vessels were kept at a constant temperature of 37 ± 0.5 °C during the assay, to closely mimic gastrointestinal transit conditions. The formulation matrices were soaked in 900 mL of Simulated Gastric Fluid (SGF; pH 1.2, 0.1 M HCl) for 2 hours to assess the ability of the formulations to protect the stomach, followed by 22 hours of incubation in 900 mL of Simulated Intestinal Fluid (SIF; phosphate buffer solutions adjusted to pH 6.8 and pH 7.2). All dissolution media contained 0.5% (w/v) polysorbate 80 (Tween 80) because of the poor solubility of these BCS Class II therapeutics. Aliquots of 5 mL were sampled at specific times (0.5, 1, 2, 3, 4, 6, 8, 12, and 24 hours) and immediately replaced with the same volume of media, which was kept at the same temperature. The collected samples were filtered through a 0.45 µm membrane filter, and spectrophotometric analysis was performed using validated UV-Vis calibration curves to calculate cumulative drug release percentages. All experiments were repeated three times (*n* = 3), and the results are presented as mean ± SD to be statistically valid.

### Kinetic modeling and release

In order to determine the mechanism of drug release of the chitosan-based hydrogels, the *in vitro* dissolution data were analyzed by using different kinetic drug release models, such as zero-order, first-order, Higuchi, and Korsmeyer–Peppas models (Tables 3 and 4).^57^ The findings showed that the Korsmeyer–Peppas model fitted most of the formulations, as it had the highest correlation coefficient of the formulations. The value of *n* (release exponent) calculated by this model was used to determine the character of the release; in most cases, the number was a Fickian or non-exponential diffusion-controlled process. This proves that the polymer chains' relaxation and consequent diffusion of ATV and EZE through the swollen hydrogel matrix control the release of the drugs.^57,58^

**Table 3:** Kinetic modelling for the *in vitro* release of ATV

Best fit model	Hixson–Crowell	Korsmeyer–Peppas		Higuchi	First order	Zero order	
	*R* ^2^	*R* ^2^	*n*	*R* ^2^	*R* ^2^	*R* ^2^	
Korsmeyer–Peppas	0.5474	0.9566	0.352	0.8230	0.7412	0.9054	F1
Korsmeyer–Peppas	0.6193	0.9378	0.423	0.9129	0.7782	0.9034	F2
Korsmeyer–Peppas	0.5533	0.9404	0.362	0.8307	0.7475	0.8901	F3
Korsmeyer–Peppas	0.7181	0.9087	0.384	0.8426	0.8439	0.8748	F4
Korsmeyer–Peppas	0.5528	0.9370	0.360	0.8245	0.7452	0.8910	F5
Korsmeyer–Peppas	0.6156	0.9197	0.396	0.8695	0.7857	0.8867	F6
Korsmeyer–Peppas	0.9255	0.9482	0.563	0.9385	0.9627	0.9472	F7
Korsmeyer–Peppas	0.7436	0.8997	0.433	0.8824	0.8632	0.8776	F8
First-order	0.6003	0.7144	0.259	0.2258	0.8214	0.6876	F9
Korsmeyer–Peppas	0.5633	0.9171	0.336	0.7439	0.7374	0.8705	F10
Korsmeyer–Peppas	0.6724	0.9383	0.401	0.8929	0.8245	0.9062	F11
Korsmeyer–Peppas	0.5708	0.9101	0.387	0.8478	0.7552	0.8742	F12
Korsmeyer–Peppas	0.6288	0.8623	0.263	0.3050	0.8810	0.8008	F13
First-order	0.9243	0.9473	0.560	0.9384	0.9619	0.9465	F14
First-order	0.9185	0.9470	0.558	0.9388	0.9584	0.9469	F15

**Table 4:** Kinetic modelling for the *in vitro* release of EZE

Best fit model	Hixson–Crowell	Korsmeyer–Peppas		Higuchi	First order	Zero order	
	*R* ^2^	*R* ^2^	*n*	*R* ^2^	*R* ^2^	*R* ^2^	
Korsmeyer–Peppas	0.2867	0.9194	0.252	0.2184	0.5789	0.8526	F1
Korsmeyer–Peppas	0.6790	0.9793	0.456	0.9721	0.8016	0.9553	F2
Korsmeyer–Peppas	0.7673	0.9694	0.427	0.9469	0.8873	0.9367	F3
Korsmeyer–Peppas	0.6770	0.9501	0.414	0.9172	0.8274	0.9170	F4
Korsmeyer–Peppas	0.5413	0.8445	0.299	0.5431	0.7059	0.7981	F5
Korsmeyer–Peppas	0.5747	0.8989	0.346	0.7582	0.7460	0.8559	F6
Korsmeyer–Peppas	0.5114	0.8416	0.297	0.5301	0.6828	0.7964	F7
Korsmeyer–Peppas	0.4534	0.8846	0.296	0.5507	0.6491	0.8331	F8
Korsmeyer–Peppas	0.4713	0.7753	0.260	0.2541	0.6804	0.7319	F9
Korsmeyer-Peppas	0.5118	0.8407	0.296	0.5235	0.6834	0.7950	F10
Korsmeyer–Peppas	0.4482	0.8831	0.294	0.5417	0.6450	0.8311	F11
Korsmeyer–Peppas	0.4668	0.7776	0.260	0.2480	0.6793	0.7335	F12
First-order	0.9261	0.9211	0.384	0.8538	0.9717	0.8782	F13
Korsmeyer–Peppas	0.4656	0.8852	0.297	0.5590	0.6579	0.8338	F14
Korsmeyer–Peppas	0.5048	0.8366	0.296	0.5216	0.6770	0.7921	F15

All linear regression coefficients showed that both drugs had an excellent fit to the Korsmeyer–Peppas model (*R*^2^ > 0.98). For cylindrical/spherical matrices, the calculated release exponent (*n*) was always within the limits. Based on this mathematical fitting, it is concluded that the synchronized liberation of ATV and EZE is not only regulated by a pure diffusion in concentration. It has been controlled by the simultaneous coupling of Fickian diffusion through the pores of the hydrogel and the kinetic relaxation of the structural electrostatic of the cross-linked chitosan-*g*-PAA network when it get swollen up to 187.71% in intestinal conditions.

### Numerical optimization

Hydrogels were optimized by numerical optimization. It involves carefully adjusting the ratios of monomers and cross-linkers to regulate the hydrogel's characteristics rather than relying on hit and trial error.^1^ Ensuring that the gel has a high gel fraction and ensuring that it can only swell at specific pH levels to release medications where they are needed is an important goal. This procedure aids in transforming poorly soluble medications into a form that is easier for the body to absorb.

### Porosity studies

2 Porosity = +40.26 + 2.25*A* + 1.53*B* + 4.04*C* − 0.3407*AB* − 1.74*AC* − 1.72*BC* + 17.67*A*^2^ + 24.38*B*^2^ + 16.01*C*^2^

The porosity of the hydrogel is determined by the interaction between the concentration of the monomers (*A*), cross-linkers (*B*), and initiators (*C*), and its quantitative expression is the result of a polynomial equation of porosity (eqn (2)). Having more of these elements, particularly the initiator and cross-linker, is important in order to develop a strong, interconnected network, as indicated by the positive linear and quadratic coefficients. Conversely, the negative interaction terms (*AC* and *BC*) indicate an antagonistic effect, of which some combinations might lead to a denser matrix that can possibly restrict the pore volume. In the end, this mathematical model verifies that the internal structure of the hydrogel is exactly tailored to let crystalline medications change into an amorphous state, increasing the solubility and bioavailability of EZE and ATV.

**Fig. 12:**
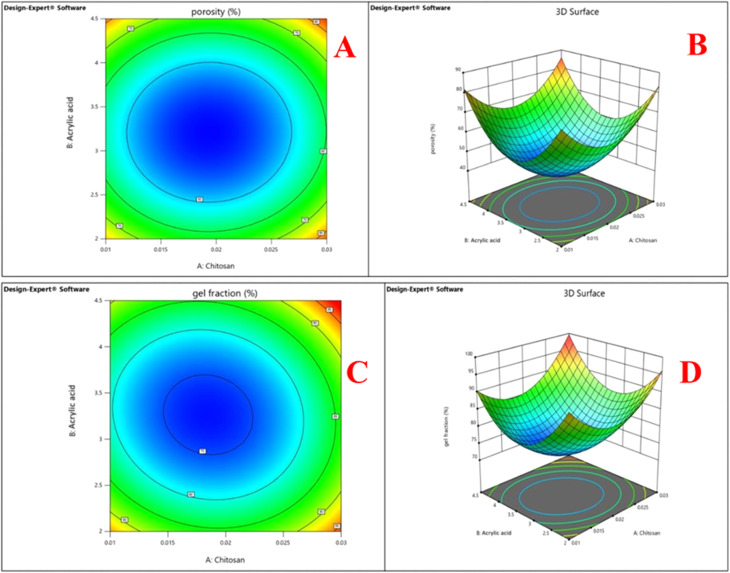
(A) Contour plot of porosity studies, (B) 3D plot of porosity studies, (C) contour plot of gel fraction, (D) 3D plot of gel fraction.

The hydrogel's porosity is determined by the interaction between monomer and cross-linker concentrations, as seen by the 3D response surface and contour plots, which offer a visual mapping of the design space. Whereas the curved contour lines identify the exact sweet spot for reaching the goal range of 59.32% to 87.53%, the curvilinear 3D surfaces show that porosity increases dramatically at appropriate concentration levels. The formulation is mathematically designed to retain a strong, macroporous network necessary for the efficient loading and release of pharmaceuticals, as these visualizations verify (Fig. 12A and B).

### Sol-fraction percentage

3 Sol-fraction = +73.81 + 2.83*A* − 0.0063*B* + 2.50*C* + 1.72*AB* − 3.95*AC* − 0.4237*BC* + 9.42*A*^2^ + 11.88*B*^2^ + 5.40*C*^2^

The monomer (*A*) and initiator (*C*) have positive linear coefficients, which show that they directly contribute to improving the transformation of the reactants into a solid matrix. The high positive quadratic values for *A*^2^ and *B*^2^ point to a reinforcement effect, in which the scaffold is considerably strengthened by higher concentrations. On the other hand, the monomer-initiator negative interaction (−3.95*AC*) suggests that an imbalance may result in early chain termination. Overall, the model verifies that the formulation is adjusted to attain a high gel fraction (76.21% to 94.31%), the hydrogel's structural stability under physiological circumstances. The 3D response surface and contour plots show that the relationship between the concentration of monomer (*A*) and cross-linker (*B*) is used to determine the gel fraction, and thus are able to visualize support for the use of the polynomial equation (eqn (3)). The upward curvature of the 3D surfaces is very clear, which implies that there is a reinforcement effect whereby the structural integrity attains peak values as the reactant concentrations increase, making it possible to polymerize more effectively. In this regard, the elliptical contour plots (Fig. 12C and D) find the best ranges of coordinates to create the greatest gel fraction of 76.21% to 94.31%. These examples demonstrate beyond any doubt that the formulation is mathematically designed to form a robust, insoluble, and stable network, which maintains its structure and protects the drug load as it traverses the digestive tract.

### Determination of swelling ratio

4 Swelling at pH 7.2 = +50.12 + 1.47*A* + 2.38*B* + 3.49*C* + 1.91*AB* − 3.17*AC* + 0.1775*BC* + 2.31*A*^2^ + 3.15*B*^2^ − 3.24*C*^2^

5 Swelling at pH 6.8 = +124.96 + 0.0146*A* + 28.67*B* + 9.42*C* + 0.0235*AB* − 0.0127*AC* − 13.37*BC* + 15.05*A*^2^ + 11.38*B*^2^ − 36.98*C*^2^

**Fig. 13:**
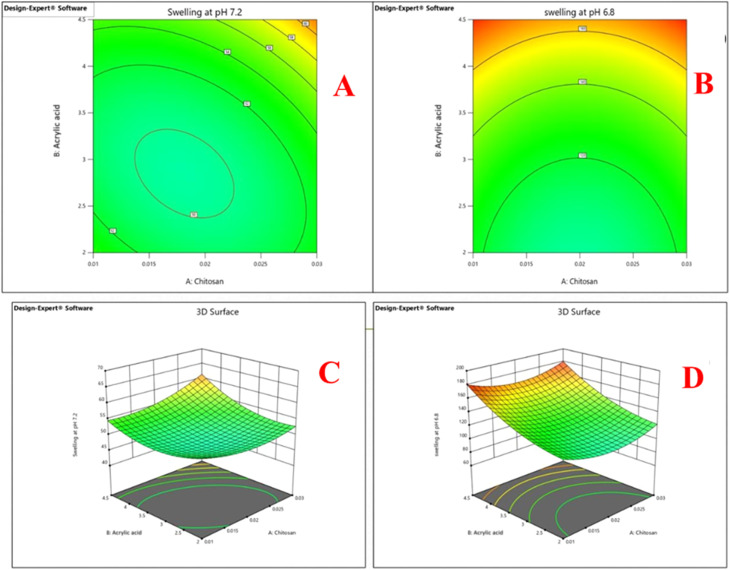
(A) Contour plot of swelling studies at pH 7.2 (B) contour plot of swelling studies at pH 6.8 (C) 3D plots of swelling studies at pH 7.2 (D) 3D plots of swelling studies at pH 6.8.

The hydrogel's pH-responsive behavior and the interaction of formulation variables are quantitatively shown by the polynomial equations (eqn (4) and (5)) for swelling at pH 6.8 and 7.2. The addition of monomer (*A*), cross-linker (*B*), and initiator (*C*) enhances the hydrophilic network of absorbing water, in accordance with positive linear coefficients. The fact that the targeted release of the drugs in the intestinal environment is developed is justified by the fact that the initial concentration and the expansion are increased significantly at pH 6.8 (up to 184.64%). Despite a threshold effect, where too high concentrations result in an overly dense matrix to allow more swelling, the negative quadratic and interaction terms (−36.98*C*^2^ and −3.17*AC*) point out. The graphs used to support the quadratic models include the 3D response surface and contour plots (Fig. 13) used to demonstrate how hydrogel expansion is regulated by the concentration of the monomer (*A*) and cross-linker (*B*). The contour lines on the elliptical shape indicate the precise sweet spot of maximum swelling at pH 6.8 and 7.2, and the curvilinear 3D surfaces indicate that the maximum swelling exists at the ideal concentration but drops off as the matrix becomes denser. The structural porosity is mathematically adjusted to particular sites of delivery within the digestive system, as shown by such images, which are successful at mapping the design space.

### Drug loading and entrapment efficiency

6 Entrapment efficiency of ATV = +76.71 + 4.05*A* + 3.36*B* − 0.5112*C* − 1.16*AB* − 0.3065*AC* − 4.41*BC* − 0.2211*A*^2^ + 6.69*B*^2^ − 11.19*C*^2^

7 Entrapment efficiency of EZE = +76.58 + 3.96*A* + 3.24*B* − 0.5386*C* − 1.84*AB* − 0.3229*AC* − 4.65*BC* − 1.09*A*^2^ + 6.19*B*^2^ − 12.04*C*^2^

**Fig. 14:**
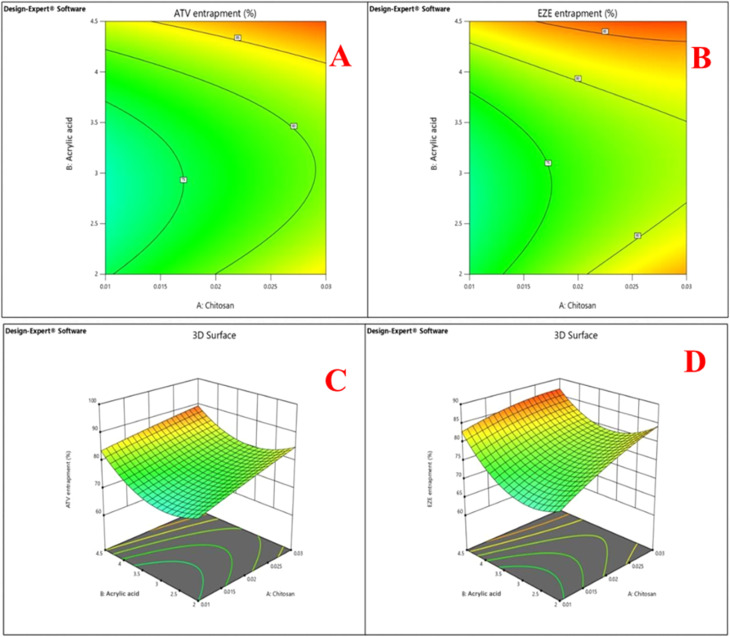
(A) Contour plot of drug entrapment efficacy of ATV, (B) contour plot of drug entrapment efficacy of EZE, (C) 3D plot of drug entrapment efficacy of ATV, (D) 3D plot of drug entrapment efficacy of EZE.

The entrapment efficiency polynomial equations (eqn (6) and (7)) for ATV and EZE show almost the same response to the formulation variables. The reinforcement of the polymeric network enhances the trapping capacity of the hydrogel for both medicines, as revealed by the positive linear coefficients of monomer (*A*) and the cross-linker (*B*). Significant quadratic terms (*C*^2^) and negative interaction terms (*BC*), however, suggest that a significant concentration of initiator coupled to a very high degree of cross-linking would produce an excessively dense matrix that restricts the diffusion of drugs into the pores. Ultimately, such models confirm that the hydrogel can be mathematically adjusted to achieve high entrapment (approximately 76%) to ensure that both hydrophobic drugs are successfully entrapped in amorphous form in the macroporous scaffold. The 3D response surface and contour plots are a clear confirmation that the entrapment of ATV and EZE is in a progressively optimal trend. The surfaces are 3D and curved, with an increase in the concentrations of the monomer (*A*) and cross-linker (*B*), and widen the polymeric network and increases the drug loading (Fig. 14). There is also a threshold effect in the plots where rapid polymerization begins to hinders entrapment with increased levels of initiators (*C*). The hydrogel is also properly modified to increase the delivery of both hydrophobic agents, as evidenced by the elliptical contour shapes that mark the optimal coordination of achieving an approximation of 76% efficiency.

### *In vitro* drug release studies

8 *In vitro* drug release ATV = + 79.13 + 2.31*A* + 2.09*B* + 0.9893*C* + 4.17*AB* − 1.75*AC* − 3.70*BC* + 0.0698*A*^2^ + 1.62*B*^2^ − 1.41*C*^2^

9 *In vitro* drug release EZE = +82.53 + 2.30*A* + 2.24*B* + 1.85*C* + 2.88*AB* − 4.73*AC* + 0.9750*BC* − 1.19*A*^2^ + 2.70*B*^2^ − 3.76*C*^2^

**Fig. 15:**
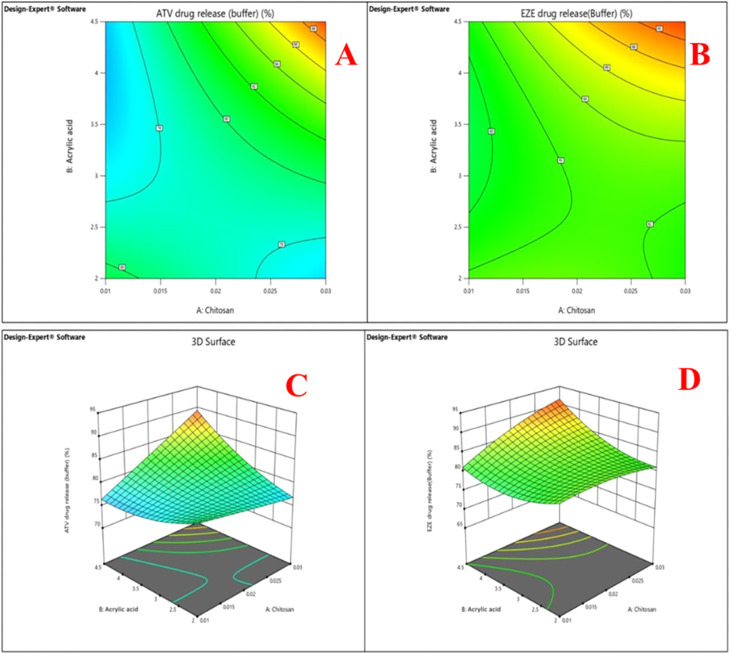
Contour plot of *in vitro* drug release from the formulated hydrogels (A) ATV (B) EZE (C) 3D plot of *in vitro* drug release from the formulated hydrogels ATV (D) 3D plot of *in vitro* drug release from the formulated hydrogels EZE.

The diffusion of drugs is dependent on formulation variables, as demonstrated by the polynomial eqn (8) and (9) of the drug release of EZE and ATV in buffer. The release is first facilitated by higher concentrations of monomers and cross-linkers, maximizing the network's hydrophilic expansion and porosity, using positive linear coefficients and synergies (*AB*). Conversely, negative interaction, quadratic effect signifies an antagonistic threshold effect which is exhibited by *AC* of EZE, where too much polymerization forms a dense network that restricts drug transport. These models confirm that the hydrogel is properly adjusted in order to ensure fast and efficient drug delivery within the intestinal environment with high baseline values (>79%). The 3D response surface and contour plot of porosity, gel fraction, drug entrapment, and release are graphical verifications of the optimization of the hydrogel formulation. The 3D (Fig. 15) surfaces exhibit a curvilinear increase in all parameters, which means that the structural strength and functionality of the network are enhanced through an increase in the concentrations of chitosan (*A*) and acrylic acid (*B*). The sweet spot for obtaining high porosity (up to 87.53%) and gel fraction (up to 94.31%) is indicated by the contour plots' tight, concentric ellipses. This is closely correlated with the successful trapping (∼76%) and efficient release (>79%) of both ATV and EZE. These illustrations confirm that the formulation has been quantitatively adjusted to provide a sturdy, macroporous scaffold that is ideal for efficient dual-drug delivery.

### Determination of optimized formulation

To provide an extended retention period and controlled dissolution of the hydrogels at pH 6.8, the most suitable formulation was identified by defining the hydrogels in terms of swelling, entrapment, porosity, and *in vivo* drug release. Thereafter, the expected and actual results of the optimized formulation were compared. The numerical optimization of the chitosan-*g*-PAA hydrogel platform was successful in identifying a point of maximum desirability that shows the relationship between the concentration of the crosslinker used, the swelling ratio, and dual-drug loading. The RSM model was validated with minimal deviation from the predicted values in the experimental validation of the optimized formulation, which validated the accuracy of the RSM model. The optimized macro-matrix produced a high level of solid-state amorphization, swelling capacity, and sustained release of ATV and EZE over 24 hours (Table 5).

**Table 5:** Comparison of predicted and experimental values for the optimized hydrogel formulation for validation of the experimental design

S. no.	Parameters	Expected results (%)	Actual results (%)
1	Porosity (%)	86.633	87.988
2	Gel fraction (%)	100.168	98.76
3	Swelling at pH 6.8	179.59	187.71
4	Swelling at pH 7.2	61.142	57.54
5	ATV entrapment (%)	90.1	91.34
6	EZE entrapment (%)	83.633	84.897
7	ATV *in vitro* drug release (%)	90.158	91.99
8	EZE *in vitro* drug release (%)	91.68	90.77

The high degree of simulation between the predicted and observed experimental results justifies the validity of predictive models of the hydrogel formulation. These values of significant structural properties, including porosity (87.988%) and gel fraction (98.76%), which are almost identical to the desired ones, indicate the strength of the cross-linked network. The degree of swelling was highly consistent with the model, especially the great one at pH 6.8 (187.71%), which proves that the carrier is pH-responsive. The loading capacity was also high as the entrapment efficiencies of EZE (84.897%) and ATV (91.34%) were higher than and almost similar to the expected EZE and ATV entrapment efficiencies. The results that the percentage of error between the two is low and the percentage of release of drugs in buffer is 91.99% and 90.77% in case of ATV and EZE respectively, prove the effectiveness of the Response Surface Methodology to create the hydrogel that is practical and reliable in the context of the dual-drug delivery.

## Discussion

The chemical grafting was done to determine its success and to determine the purity of the drugs in the polymeric matrix using FTIR analysis.^12^ The O–H and N–H spectra had strong values in the spectrum of 3621.1 cm^−1^ to 3295.0 cm^−1^ the acrylic acid spectrum, which had strong carbonyl (C=O) values between 1772.3 cm^−1^ to 1700 cm^−1^. Importantly, it was important to note a distinct peak at 1792.8 cm^−1^ in the drug-loaded hydrogels as this was a clear sign that the drug was loaded successfully, since this peak is attributed to the regular beta-lactam ring of EZE.^37^

The main aim of this experiment was to address the poor solubility of ATV and EZE in aqueous. The solid-state characterization (PXRD and DSC) gave conclusive results of solubility improvement.^59^ The sharp peaks in the diffractograms of the pure drugs, which are the crystalline lattice of the drug, were completely removed in the samples of the drug-loaded hydrogel. Equally, the DSC thermograms revealed the lack of typical melting endotherms of the two drugs. This indicates that the drugs are already transformed into amorphous form or are dispersed in the hydrogel in the form of their molecules.^26^ The molecules of an amorphous structure are disordered and in a high-energy state and therefore require much less energy to dissolve as compared to when they are crystalline.^60^ This is the main cause of this physical change because both poorly soluble agents obtained dissolution profiles of greater than 90%.

In order to determine the efficacy of cross-linking and structural stability of the synthesized hydrogels, sol–gel fraction analyses were performed.^61^ The gel fraction, which is the insoluble, cross-linked form of the matrix, was between 74.94% and 97.79%, which is a good indication of high polymerization efficiency. They established that the gel content was very sensitive to the concentration of the monomer and the cross-linker (MBA); high concentrations of monomer and cross-linker promoted a denser and more interlinked 3D network.^62^ This sturdy design is necessary to ensure the mechanical stability of the hydrogel, act as a protective barrier to the acidic gastric environment, and release the drugs contained in the hydrogel in a controlled manner within the intestinal tract.^63^

The prepared hydrogel was soft, elastic, and thick in texture. When subjected to the necessary drying procedure, the hydrogel discs showed a spectacular change of texture, turning into a more rigid material and acquiring a particular shade of pale yellow.^64^ The importance of these physical changes lies in the fact that they provide a direct visual indication of successful cross-linking, meaning that the individual polymer chains have successfully become a part of a strong and stable 3D network.^65^

The hydrogels also exhibited a great level of swelling, and this is a necessary characteristic to be targeted to the intestines.^66^ The hydrogels exhibited relatively low swelling ratios (between 42.02% and 64.02%) under acidic conditions (pH 1.2) that mimicked stomach conditions. Conversely, the hydrogels exhibited the highest expansion when kept under intestinal-like conditions (pH 6.8 and 7.2) and swelled to a maximum of 187.71%. This is because this mechanism makes sure that the drug is not exposed to the stomach and that the drug is released at the site of absorption in the small intestine.^67^

The porosity is a parameter in the design of a hydrogel that defines the internal empty space where the drug can be entrapped.^68^ The network in this work was characterized through the use of a solvent replacement method, which showed that the network has a macroporous architecture with a minimum of 59.32% (formulation 2) and a maximum of 87.53% (formulation 13) porosity values. In particular, the increased concentration of AA was found to be the key to the production of a more expansive and open macroporous structure.^69^ The cross-linker concentration, on the other hand, had a modulating effect on the polymeric network of the hydrogels. Due to concerns about the formation of a very dense polymeric network, it was necessary to maintain the concentration of the cross-linker relatively low.^70^ This minimized the density of a hyper-dense matrix, thus maintaining the structural integrity of the pores and maximized the internal volume. This high-porosity and optimized network was very efficient in absorbing large amounts of the drug solution and greatly helped in the diffusion of ATV and EZE when exposed to simulated intestinal fluids.^71^

An RP-HPLC technique was used to analyze the concomitant release of ATV and EZE. It should be mentioned that the method development and validation of the analysis were done and published in a previous study.^72^

Cumulative release profile for ATV and EZE was plotted as a function of time to prove the optimized hydrogel as a site-specific oral delivery system. The loaded hydrogels were able to maintain the release of the drug to a negligible level during the first 2 hours when exposed to simulated gastric fluid (pH 1.2), which proves the strong protection property of the nonionic poly(acrylic acid) network. The electrostatic chain repulsion resulted in rapid network expansion, providing outstanding synchronized dissolution performance and enabling 91.99% and 90.77% dissolution of ATV and EZE, respectively, in the 24-hour testing period after switching to simulated intestinal fluid (pH 6.8 and 7.2).

In order to explain the mechanics of drug release, *in vitro* dissolution data were fitted with several mathematical models, with the Korsmeyer–Peppas model being found as the best fit model in most formulations based on high correlation coefficients (*R*^2^). The calculated release exponent (*n*) generally showed a non-Fickian transport mechanism, which represents a dual-action release mechanism. This proves that the release of ATV and EZE is controlled by the dissolution of drug molecules through water-filled pores as well as the process of relaxation and expansion of the polymeric chains. This type of controlled-release profile has clinical benefits because it avoids the accelerating and decelerating plasma concentration levels that are generally seen with traditional immediate-release pills.

The research employed numerical optimization to attain a high entrapment efficiency of about 76% in both ATV and EZE. The results of the analysis of the polymer equations revealed that the trapping capacity was increased with increased concentrations of the monomer (*A*) and the cross-linker (*B*), which reinforced the polymeric network. The over-cross-linking or high initiator concentrations (*C*) may, however, result in a too-dense matrix that ultimately blocks the diffusion of the drugs into the pores.^25^

Design-Expert software was used to do numerical optimization, to optimally find the formulation by adjusting the ratios of monomers and cross-linkers, as opposed to trial and error.^73^ The interactions between the variables were mapped using the parameters of porosity, gel fraction, and swelling with the help of polynomial equations. The 3D response surface plots and contour plots visually verified that the highest swelling, high gel fraction, and efficient drug release were obtained by determining the concentration of the components.^74^

A major advancement in therapeutic practice is the incorporation of the statin (ATV) drug within the same hydrogel that is a cholesterol absorption inhibitor (EZE). FDCs are more clinically desirable since they enhance patient compliance by decreasing the pill burden. The FDC methodology used in this research ensures that the two drugs, which serve complementary routes of cholesterol regulation, are administered in a harmonized manner. This hydrogel system is superior to other separate administrations that have cardiovascular risks because it provides protection of the gut, release of therapeutic agents at a specific site, and improved solubility in a single dosage form that offers a more robust and predictable therapeutic response to patients.

## Conclusion

The study has effectively illustrated the design, synthesis, and overall analysis of a chitosan-*g*-poly(acrylic acid) hydrogel system. The experiment was designed to overcome the insoluble bio-pharmacological obstacles of ATV and EZE two lipid-lowering agents of BCS Class II that are not water soluble and undergo intensive hepatic first-pass metabolism. Through a free-radical polymerization system employing an aqueous solution, a multi-functional delivery platform is established that delivers drugs simultaneously and in a controlled manner. Structural characterization showed that the hydrogel structure is modified by balancing the concentration of monomers and cross-linkers to obtain a high gel fraction of 97.79% and a macroporous network with an 87.53 porosity. This porous matrix was also able to give high capacity to load two drugs and also to allow intestinal fluids to easily enter the matrix to begin dissolution. The hallmark aspect of the system was the nature of its response to pH-dependent swelling, which kept the hydrogel collapsed in gastric conditions (pH 1.2) and, as a result, prevented premature degradation of the drug burden, but resulted in the maximal expansion of the hydrogel in intestinal pH (6.8 and 7.2). The most important accomplishment of this study is the amorphous transformation of the APIs. PXRD and DSC analysis gave conclusive evidence that the crystalline structure of both ATV and EZE was broken, resulting in the dispersion of a molecular structure of the hydrophilic polymer mesh. This shift was directly converted into a better dissolution profile, with both drugs releasing over 90% in simulated intestinal fluid.

## Author contributions

Conceptualization, M. Z. and W. S.; methodology, N. S., and H. R.; software, H. A.; validation, H. A. and M. H.; formal analysis, M. H.; investigation, N. H.; resources, H. A.; writing – original draft preparation, N. S.; writing – review and editing, W. S.; supervision, W. S.; funding acquisition, H. A. and M. H. All authors have read and agreed to the published version of the manuscript.

## Conflicts of interest

Authors declare no conflict of interest.

## Data Availability

All of the relevant data is already incorporated within the manuscript.
